# Comparison of strategies for identification of regulatory quantitative trait loci of transcript expression traits

**DOI:** 10.1186/1753-6561-1-s1-s85

**Published:** 2007-12-18

**Authors:** Nora Franceschini, Mary K Wojczynski, Harald HH Göring, Juan Manuel Peralta, Thomas D Dyer, Xia Li, Hao Li, Kari E North

**Affiliations:** 1 Department of Epidemiology, University of North Carolina Chapel Hill, Bank of America Center, 137 East Franklin Street, Suite 306, CB #8050, Chapel Hill, North Carolina 27514, USA; 2 Department of Biostatistics, University of Alabama-Birmingham, 1665 University Boulevard, Ryals Public Health Building 414, Birmingham, Alabama 35294, USA; 3 Southwest Foundation for Biomedical Research, PO Box 760459, San Antonio, Texas 78245-0549, USA; 4 Glaxo-Smith-Kline, 17.2136A Sanders Building, 5 Moore Drive, Research Triangle Park, North Carolina 27709, USA

## Abstract

In order to identify regulatory genes, we determined the heritability of gene transcripts, performed linkage analysis to identify quantitative trait loci (QTLs), and evaluated the evidence for shared genetic effects among transcripts with co-localized QTLs in non-diseased participants from 14 CEPH (Centre d'Etude du Polymorphisme Humain) Utah families. Seventy-six percent of transcripts had a significant heritability and 54% of them had LOD score ≥ 1.8. Bivariate genetic analysis of 15 transcripts that had co-localized QTLs on 4q28.2-q31.1 identified significant genetic correlation among some transcripts although no improvement in the magnitude of LOD scores in this region was noted. Similar results were found in analysis of 12 transcripts, that had co-localized QTLs in the 13q34 region. Principal-component analyses did not improve the ability to identify chromosomal regions of co-localized gene expressions.

## Background

There is a breadth of information being generated by the Human Genome Project and the interpretation of these data has been a major area of research. For simple Mendelian disorders, the identification of genetic effects is fairly straightforward due to understanding the biology that drives these disorders. However, for complex oligogenic or polygenic disorders, understanding all the interconnections between genes influencing a trait is a difficult task because the understanding of the biology of many of these disorders is still evolving. Multiple gene × gene and gene × environment interactions can influence the expression of phenotypes. Genes can interact by modifying the expression of other genes and therefore function as regulatory genes [1].

In an effort to dissect some of these complexities, we performed linkage analysis of gene expression transcripts of members of Centre d'Etude du Polymorphisme Humain (CEPH) Utah families to determine the heritability of transcripts and the evidence for regulatory quantitative trait loci (QTLs) and we performed pairwise bivariate linkage analysis and principal-component analysis (PCA) for data-reduction to evaluate the evidence for shared genetic effects. The ability to assess gene expression traits simultaneously and to link them to QTLs offers the possibility of identifying previously unknown underlying molecular processes for future investigation.

## Methods

### Population and phenotypes

We used the Genetic Analysis Workshop 15 (GAW 15) Problem 1 microarray gene expression profiles for the analyses. Data were available for 14 three-generation CEPH Utah families. Expression levels of genes were obtained from lymphoblastoid cells using the Affymetrix Human Focus Arrays [2]. We were provided with data on 3554 transcripts that showed greater variation between individuals than within the same individual.

Family members were genotyped for 2882 autosomal and X-linked single-nucleotide polymorphisms (SNP) generated by the SNP Consortium . Genetic map positions were obtained using the SNP Mapping web application developed by the University College Dublin Conway Institute of Biomolecular and Biomedical Research , which uses the Rutgers Combined Linkage-Physical Map of the Human Genome and data taken from the NCBI dbSNP Build 123 (in Kosambi centimorgans). This information was used to calculate multipoint identity by descent matrices (MIBDs) with Merlin and Minx [3], after removal of Mendelian inconsistencies and double recombinants with Preswalk (based on Simwalk mistyping probabilities) [4]. MIBDs were used for linkage analyses.

Transcript distributions were normalized using an inverse normalization transformation of z-scores of individual transcripts regressed on the mean individual transcript level. We further adjusted for the effects of age, age^2^, sex, age × sex and age^2 ^× sex interaction using predictive linear regression models in SAS 9.1 (Cary, NC). We generated these residuals as part of our processing of the transcripts for linkage analyses.

### Heritability estimation and linkage analysis

Heritability was estimated using maximum likelihood variance decomposition methods in *SOLAR *[5,6]. Genome scans were performed using multipoint variance-components models that test for linkage between traits and genetic variants by partitioning the variance of the expression level into its additive genetic and environmental variance components [7]. For transcripts with co-localized QTLs, we performed bivariate linkage analysis to identify shared genetic effects. The bivariate polygenic model estimates correlations caused by residual additive genetic effects (ρ_G_) and correlations caused by random environmental effects (ρ_E_) [8]. To test for additive genetic correlation among pairs of traits, the log likelihood of a model in which ρ_G _is constrained to 0 (null hypothesis, no correlation) or ρ_G _= 1 (null hypothesis, complete shared genetic effect) is compared to that of a model in which ρ_G _is estimated for the traits. Significant differences among the models (ρ_G _≠ 0) suggest that some of the same genes influence both traits. We also performed linkage analysis using the factors obtained from the PCA in a sample of transcripts with co-localized QTLs.

### Principal-component analysis

PCA was used to reduce the number of expression profiles into statistically meaningful groups while retaining the original total variance using all the expression profiles [9,10]. We selected two different chromosomal regions of a length of 10 to 12 MB in which the QTLs of at least 10 transcripts were co-localized. Only transcripts of genes that were not located in these selected chromosomal regions were included in the analyses (*trans*-regulatory genes). Because of the small number of individuals in the study and concerns of overfitting the model, a maximum sample of 50 transcript values were considered at one time [11]. The number of factors was determined using the eigenvalue-one requirement [11]. Factors are interpreted by examining the varimax-rotated factor loadings, which are the correlations between each phenotype and the factor in question. Factor loadings greater than or equal to 0.40 in absolute value were used to interpret factors and to characterize the factor structures; this criterion ensures that the individual factor variables share at least 15% of their variance with the given factor [9]. The principle components were obtained by calculating the eigenvalues of the sample covariance matrix, which represent the amount of variance contributed by each factor. Only factors with eigenvalues higher than 1 were considered for linkage analysis.

### Integrating data from linkage analysis for gene co-expression

Linkage signals of individual transcript expressions were recorded and the location of QTLs was compared to the location of the transcript gene in order to identify *trans*-regulatory sites. In addition, the location and LOD scores of QTLs identified in single individual transcript analysis (univariate analysis) were compared with the location of the QTLs identified using bivariate analysis or factors of the PCA. This allowed a determination of whether the bivariate analysis or PCA data reduction analysis improved our evidence for linkage, and if so, a more in-depth examination of the transcripts included in the principal components needs to be examined for biologic interactions on complex disorders.

## Results

Among 194 individuals from 14 families, 17 individuals with missing information on age were excluded. Seventy-six percent (*n *= 2688) of the transcripts had significant heritability (*p *< 0.05) and were considered for the linkage analysis. Of this, 1448 (54%) transcripts displayed suggestive evidence of linkage (had a maximum LOD score ≥ 1.8 [12]). The QTLs of 1661 transcripts (759 of which with LOD ≥ 1.8) were localized in a different region than the gene transcript (*trans*-regulatory sites). We used two different chromosomal regions with co-localized transcript QTLs, chromosomes 4q28.2-q31.1 and 13q34, for more in-depth analyses.

### Chromosome 4q28.2-q31.1 region

Table 1 reports the results for the chromosome 4q28.2-q31.1 region. Fifteen transcripts co-localized in this region in the univariate linkage analysis, and the LOD scores ranged from 1.17 to 3.72. The strongest linkage signals were observed for the transcripts of the *MX2*, *NUCB2*, and *SNX4 *genes. Using PCA, we obtained five factors from the 15 transcripts with eigenvalues greater than 1. Only one factor, with a high positive loading for the *MX2 *gene transcript, had a significant heritability and a LOD score ≥ 1.8. The linkage analysis using this factor identified the chromosome region for the *MX2 *gene, but the LOD score was lower than the one obtained by single linkage analysis of the *MX2 *transcript.

**Table 1:** Univariate transcript heritability and linkage analysis compared to principal-component approach: chromosome 4q28.2-31.1 region

Transcript	Group^a^	Gene name	Gene symbol	H^2 ^(SE)^b^	H^2 ^*p*-value	LOD score	Trait locus location	Transcript gene locus
218935_at		EH-domain containing 3	*EHD3*	0.20 (0.13)	0.01	1.98	4q28.2	2p21
212652_s_at	1	sorting nexin 4	*SNX4*	0.21 (0.09)	0.001	3.03	4q28.2	3q21.2
212426_s_at	1	tyrosine 3-monooxygenase/tryptophan 5-monooxygenase activation protein, theta polypeptide	*YWHAQ*	0.40 (0.15)	<0.001	2.12	4q28.2	2p25.1
207076_s_at	1	argininosuccinate synthetase	*ASS*	0.19 (0.11)	0.01	1.17	4q28.2	9q34.1
213798_s_at	2	CAP, adenylate cyclase-associated protein 1 (yeast)	*CAP1*	0.16 (0.09)	0.008	1.73	4q28.2	1p34.2
220143_at		LUC7-like (*S. cerevisiae*)	*LUC7L*	0.16 (0.09)	0.013	1.79	4q28.3	16p13.3
204994_at	1	myxovirus (influenza virus) resistance 2 (mouse)	*MX2*	0.30 (0.13)	<0.001	3.72	4q28.3	21q22.3
200974_at		actin, alpha 2, smooth muscle, aorta	*ACTA2*	0.20 (0.09)	0.003	1.61	4q28.3-q31.1	10q23.3
201397_at	2	phosphoglycerate dehydrogenase	*PHGDH*	0.32 (0.12)	<0.001	2.12	4q28.3-q31.1	1p12
203882_at	1	interferon-stimulated transcription factor 3, gamma 48 kDa	*ISGF3G*	0.19 (0.10)	0.006	1.79	4q28.3-q31.1	14q11.2
203675_at	2	nucleobindin 2	*NUCB2*	0.47 (0.17)	<0.001	3.15	4q28.3	11p15.1-p14
201195_s_at		solute carrier family 7, member 5	*SLC7A5*	0.16 (0.09)	0.01	1.59	4q31.1	16q24.3
201681_s_at	2	discs, large homolog 5 (*Drosophila*)	*DLG5*	0.22 (0.12)	0.005	2.05	4q31.1	10q23
202531_at		interferon regulatory factor 1	*IRF1*	0.21 (0.14)	0.02	2.80	4q31.1	5q31.1
202732_at	2	protein kinase (cAMP-dependent, catalytic) inhibitor gamma	*PKIG*	0.38 (0.11)	<0.001	1.78	4q31.1	20q12-q13.1
Principal component analysis, 15 transcripts factor^c^			N/A	0.29 (0.14)	0.001	1.99	4q28.3	N/A
Group 1 factor 1 (loading *MX2*)			N/A	0.27 (0.13)	0.002	2.28	4q28.3	N/A
Group 1 factor 2 (loading *YWHAQ*)			N/A	0.29 (0.13)	0.001	0.00	4q28.2-31.1	N/A

^a^Group 1 and 2 have transcripts correlated using bivariate analysis. Transcripts without correlation on bivariate analyses were not assigned a group.

^b ^H^2^, heritability; SE, standard error; N/A, not apply.

^c ^For principal component analysis, only factors with significant heritability (alpha = 0.05) are shown.

**Figure 1:**
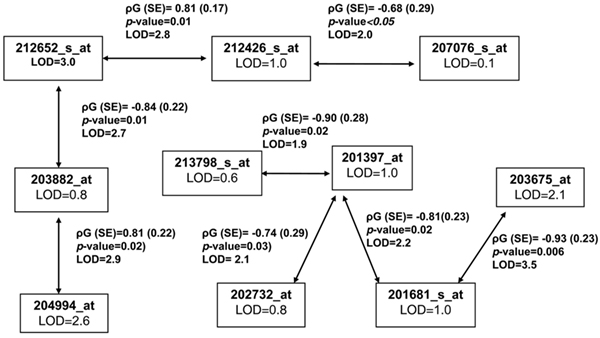
**Chromosome 4 co-localized gene transcripts univariate and bivariate analyses results (*n *= 15 transcripts)**. Each box has the transcript name (in bold) and the univariate transcript LOD score. Genetic correlation (ρ_G_) between two transcripts and *p*-values are displayed in the outside box along with the bivariate LOD scores. We found two potential networks of regulatory genes among 15 co-expressed transcripts on the 4q28.2 to 4q31.1 region. Five transcripts did not have significant genetic correlation with any other transcript and are not included in this graph.

We then performed bivariate analysis of all pairwise co-localized transcripts on 4q28.2-q31.1 and found evidence for genetic correlation of co-localized genes, although without much increase in the magnitude of the LOD score (Figure 1). This analysis identified two networks of gene expressions (Figure 1). We obtained two factors using PCA of the first network (Group 1, *SNX4*, *YWHAQ*, *ASS*, *MX2*, and *ISGF3G *gene transcripts). Both factors had significant heritability; however, only Factor 1, loading heavily on the *MX2 *gene, localized to the 4q28.2-q31.1 region (Table 1), and the magnitude of the LOD score was lower than that of the univariate *MX2 *gene transcript analysis (LOD = 2.28). The heritability of one factor obtained using PCA for Group 2 transcripts was not significant and further analysis was not performed.

### Chromosome 13q34 region

**Figure 2:**
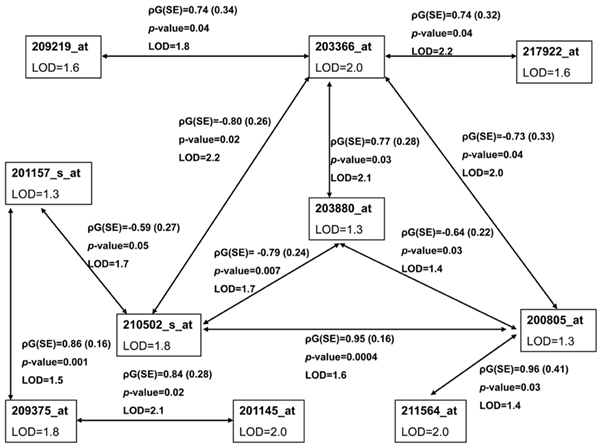
**Chromosome 13 co-localized gene transcriptsunivariate and bivariate analyses results (*n *= 12 transcripts)**. See legend to Figure 1 for explanation of symbols.

We performed analysis in an additional chromosome region of co-localized transcripts, 13q34 region, and noted similar results. Using univariate analysis, 12 transcripts co-localized in this region; and bivariate analysis revealed an intricate network of correlated traits (Table 2 and Figure 2). Using PCA, we obtained five factors, three of them with significant heritability. Similar to our previous findings on chomosome 4, PCA factors did not improve the magnitude of the LOD scores when compared to univariate analysis.

**Table 2:** Univariate transcript heritability and linkage analysis compared to principal-component approach: chromosome 13q34 region

Transcript	Gene Name	Gene Symbol	H^2 ^(SE)^a^	H^2 ^*p*-value	LOD score	Trait locus location	Transcript gene locus
200805_at	lectin, mannose-binding 2	*LMAN2*	0.25(0.10)	0.0003	1.3	13q33.2-q34	5q35.3
209375_at	xeroderma pigmentosum, complementation group C	*XPC*	0.23 (0.13)	0.007	1.8	13q33.2-q34	3p25
211564_s_at	PDZ and LIM domain 4	*PDLIM4*	0.20 (0.13)	0.01	2.0	13q34	5q31.1
203366_at	polymerase (DNA directed), gamma	*POLG*	0.21 (0.11)	0.002	2.0	13q34	15q25
210502_s_at	peptidylprolyl isomerase E (cyclophilin E)	*PPIE*	0.40 (0.13)	<0.001	1.8	13q34	1p32
217922_at	Mannosidase, alpha, class 1A, member 2	*MAN1A2*	0.21 (0.10)	0.003	1.6	13q34	1p13
209715_at	chromobox homolog 5 (HP1 alpha homolog, Drosophila)	*CBX5*	0.28 (0.11)	<0.001	2.4	13q34	12q13.13
203880_at	COX17 homolog, cytochrome c oxidase assembly protein (yeast)	*COX17*	0.28 (0.11)	0.0004	1.3	13q34	3q13.33
201145_at	HCLS1 associated protein X-1	*HAX1*	0.33 (0.14)	0.0004	2.0	13q34	1q21.3
201157_s_at	N-myristoyltransferase 1	*NMT1*	0.31 (0.11)	0.0002	2.0	13q34	17q21.31
209219_at	RD RNA binding protein	*RDBP*	0.36 (0.11)	<0.001	1.6	13q34	6p21.3
217932_at	mitochondrial ribosomal protein S7	*MRPS7*	0.11 (0.08)	0.05	1.0	13q34	17q25
Principal component analysis, factor 2 (loading *HAX1*)^b^		N/A	0.28 (0.13)	0.001	1.6	13q34	N/A
factor 3 (loading *MRPS7*)		N/A	0.21 (0.10)	0.003	1.1	13q33.1-33.2	N/A
factor 5 (loading *NMT1*)		N/A	0.20 (0.12)	0.02	1.9	13q34	N/A

^a^H^2^, heritability; se, standard error; N/A, not apply.

^b ^For principal component analysis, only factors with significant heritability (alpha = 0.05) are shown.

## Discussion

In this study, we identified co-localized QTLs of individual transcripts and compared the univariate and bivariate linkage results using single transcripts to those using factors obtained from PCA. By using factors that accounted for the variance of multiple transcripts with co-localized QTLs, we attempted to reduce the number of linkage analyses performed as well as possibly identifying previously unknown patterns of associated gene expression profiles. The PCA did in fact reduce the number of linkage analyses performed, but it did not improve the magnitude of signals in the target QTLs as compared with univariate or bivariate analyses. In fact, in at least one case, PCA was unable to detect a linkage signal for the main gene transcript loading in the factor (Table 1, Group 1, Factor 2).

We also performed pairwise bivariate genetic analysis on those transcripts that co-localized to the same genomic region, presumably because this area of the genome harbored genes involved in the regulation of these transcripts [2]. We detected significant genetic correlation of these co-localized transcripts, indicating potential gene networks operating in these regions. However, in most cases, bivariate linkage analysis did not improve the magnitude of the LOD score compared to univariate analysis. Most traits were highly correlated (ρ_G _> 0.60), and therefore they may provide redundant information that may reduce the power for detection of the bivariate signal [8]. In addition, because ρ_G _is a test of the overall additive genetic correlation among two traits and not the QTL-specific pleiotropy, it is possible that the co-localized linkage signals are not in fact genetically correlated. Further analysis is required to address these issues.

The chromosome regions selected for detailed analyses were arbitrarily chosen as we identified multiple other regions with co-localized linkage of gene expressions. The results from our univariate genome scan differ markedly from those reported by Morley et al. [2] because we included a smaller sample of individuals so that adjustment for covariate effects of age could be made. Our analysis strategy also adjusted for the effects of age and sex, which could also add to the observed differences [13]. Finally, our definition of genome window size for co-localized gene expressions was twice larger than the one described in the study of Morley et al.

## Conclusion

We identified several chromosomal regions of co-localized *trans*-regulatory genes with significant heritability. Some of these regulatory genes displayed strong additive genetic correlations, and may be part of genetic networks. However, when compared to univariate analysis, linkage analysis of bivariate phenotypes and factor scores obtained from PCA did not improve the ability to identify chromosomal regions of co-localized gene expressions.

## List of Abbreviations

CEPH: Centre d'Etude du Polymorphisme Humain

GAW: Genetic Analysis Workshop

H^2^: heritability

LOD: logarithm of the odds

MIBD: multipoint identity-by-descent matrices

N/A: not apply

NCBI: National Center for Biotechnology Information

PCA: principal-component analysis

QTL: quantitative trait loci

SE: standard error of the mean

SNP: single-nucleotide polymorphism

SOLAR: Sequential Oligogenic Linkage Analysis Routines

## Competing interests

The author(s) declare that they have no competing interests.
